# Development and stability of an in-house reference for blomia tropicalis allergen extract

**DOI:** 10.1186/1939-4551-8-S1-A174

**Published:** 2015-04-08

**Authors:** Arelis Más Quintero, Yunia Oliva Diaz, Maytee Mateo Morejón, Raysa Cruz, Mary Carmen Reyes Zamora, Raúl Lázaro Castro Almarales, Wendy Ramírez González, Damaris Torralba Averoff, Alexis Labrada

**Affiliations:** 1 National Center of Bioproducts , Cuba

## Background

Standardization of allergen vaccines is mainly based on In-House References (IHR), which are required for routine quality control. Substitution of IHR batches can be a delicate process that requires reproducibility of the reference material and detailed in-vitro characterization and in-vivo testing.

## Objective

To develop a new IHR, of the *Blomia tropicalis* standardized allergen extract and to predict its validity period through an accelerated stability study.

## Methods

Standardized allergen extract of *Blomia tropicalis* was manufactured by BIOCEN (Cuba). The batch selected for IHR was characterized by *in-vitro* allergenic activity compared to the previous IHR, using ELISA-IgE-inhibition assays, with a pool of sera from patients allergic to *Blomia tropicalis*. Allergenic and protein composition was determined by Western-Blotting-IgE and SDS-PAGE. As final criterion allergenic activity was measured by *in-vivo* Skin Prick Test in allergic patients by parallel application of three dilutions. Relative potency was calculated by the method of parallel lines. The activity was expressed in Biological Units (BU), which is related to the skin reaction size produced by Histamine 10 mg/mL. Stability was assessed by means of an accelerated study at 4 temperatures (-70°, 4°C, 37°C and 60°C) during one year, testing at 0, 3, 6, 9 and 12 months.

## Results

The physical-chemical characterization of the new IHR, complies with the limits established by national and international guidelines. A good agreement was found between the *in-vivo* and *in-vitro*, results for the allergenic potency: 100 453 UB (95%CI from 130 444 to 77 357), showing no significant difference with respect to the previous IHR. Therefore, the new IHR is equivalent to the later, regarding *in-vitro* and *in-vivo* allergenic activity, protein and allergenic composition. The IHR remained stable at -70°C, 4°C and 37°C for 12 months. At 60°C, despite a change in cake appearance, the allergenic activity was kept during the first 6 months within the specified limits.

## Conclusions

A new IHR of *Blomia tropicalis* allergen vaccine was developed, replacing the previous batch dating from 2006. It was certified for quality control use. The results predicted stability at -70°C for more than 10 years, thus assuring the reproducibility and quality of routine manufacturing batches.

